# Reduced Expression of Immune Response Genes in Neural Cells with Mutations in the PARK2 Gene in Parkinson’s Disease

**DOI:** 10.32607/actanaturae.27664

**Published:** 2026

**Authors:** V. B. Fedoseyeva, E. V. Novosadova, V. V. Nenasheva, L. V. Novosadova, I. A. Grivennikov, V. Z. Tarantul

**Affiliations:** National Research Center “Kurchatov Institute”, Moscow, 123098 Russia; Federal Research and Clinical Center for Physicochemical Medicine named after Academician Yu.M. Lopukhin, Federal Medical and Biological Agency, Moscow 119435 Russia

**Keywords:** Parkinson’s disease, induced pluripotent stem cells, glia, neural precursors, differential gene transcription

## Abstract

Parkinson’s disease (PD) is one of the most common chronic neurodegenerative diseases. PD is characterized by the dysfunction of multiple body functions caused by changes in the expression of a large number of genes. Current evidence suggests that changes in the innate immunity and neuroinflammation may play an important role in the pathogenesis of the disease. However, the exact mechanism through which immune dysfunction develops in the context of PD pathogenesis remains unclear. In this study, with the use of transcriptome sequencing (RNA-seq), followed by quantitative PCR, we managed to detect a differential expression of the genes involved in the immune activity in neural progenitors (NPs) and glial cells derived from induced pluripotent stem cells from healthy donors (HDs) and PD patients carrying mutations in the* PARK2 *gene. Expression of many of the genes involved in a number of innate immune signaling pathways (in particular, in the canonical NFκB, non-canonical NFκB, the TNFα/NFκB, IL6/STAT3, IL2/STAT5 pathways, as well as the IFNγ and IFNα response) in cells from PD patients was found to be reduced compared to that in the cells from healthy donors. A mechanism for regulating these signaling pathways in the neural precursors of PD patients carrying mutations in the *PARK2 *gene is proposed.

## INTRODUCTION

Parkinson’s disease (PD) is one of the most common neurodegenerative diseases in the world. It is estimated to affect more than one in 100 people aged 65 and over; its incidence is expected to double by 2030 [1]. In PD patients, the functions of dopaminergic and other neurons, as well as motor functions, are perturbed and immune system processes are altered [2, 3]. Although some of the risk factors and molecular mechanisms that lead to the development of PD have been identified, the pathology of this disease remains not well understood.

Neuroinflammation is a key process of innate immunity, which helps protect the brain from pathogens of various origins. However, disruption of inflamma tory processes is often accompanied by the development of neurodegenerative diseases [4, 5, 6]. In the central nervous system, microglia and astrocytes are responsible for innate immune protection, which plays a key role in neuroinflammation. A large body of data obtained from both *in vitro *and *in vivo *studies indicates that neuroinflammation, mainly mediated by microglia and astrocytes, is associated with the pathogenesis of PD [7, 8, 9, 10, 11, 12, 13, 14, 15]. There is data demonstrating that disruption of the neuron–glial interactions in PD leads to neuronal death [16, 17, 18, 19]. Dopaminergic (DA) neurons express a wide range of cytokine and chemokine receptors; so, they can be sensitive to inflammatory mediators [20]. However, it remains unclear whether changes in the immune system are a conse quence of disease onset or its cause. The changes in innate immunity, in particular inflammatory ones, in patients with PD associated with various mutations have not been sufficiently studied. Primarily, this is because of the limited availability of clinical material from patients with PD. There currently exists a wide range of experimental models employing animals and cultured cells *in vitro*, which helps obviate this limitation and study various aspects of PD [21, 22].

Induced pluripotent stem cells (iPSCs), which are obtained by reprogramming of the fibroblasts from PD patients and their neural and glial derivatives, are some of the most commonly used models of PD. In the present study, neural progenitor cells (NPs) and glia derived from iPSCs from healthy donors (HDs) and PD patients carrying various mutations in the *PARK2 *gene were used as model systems to search for differentially expressed genes (DEGs) related to the functioning of innate immunity in PD. Mutations in the *PARK2 *gene are the second-most common causes of the monogenic form of PD, which is responsible for the early onset of the disease [23]. The *PARK2 *gene encodes Parkin E3 ubiquitin ligase, which is involved in the control of substrate protein folding, mitochondrial quality assessment, and degradation of damaged mitochondria via mitophagy [8, 24].

Although there are some indications of an association between *PARK2 *dysfunction and the innate immunity in the pathogenesis of PD [15], the issue for the most part remains an open one. Our study focuses on the expression of innate immunity genes in patients carrying PD-associated mutations in the *PARK2 *gene.

## EXPERIMENTAL

**Cell lines. RNA preparation for sequencing**

**Fig. 1:**
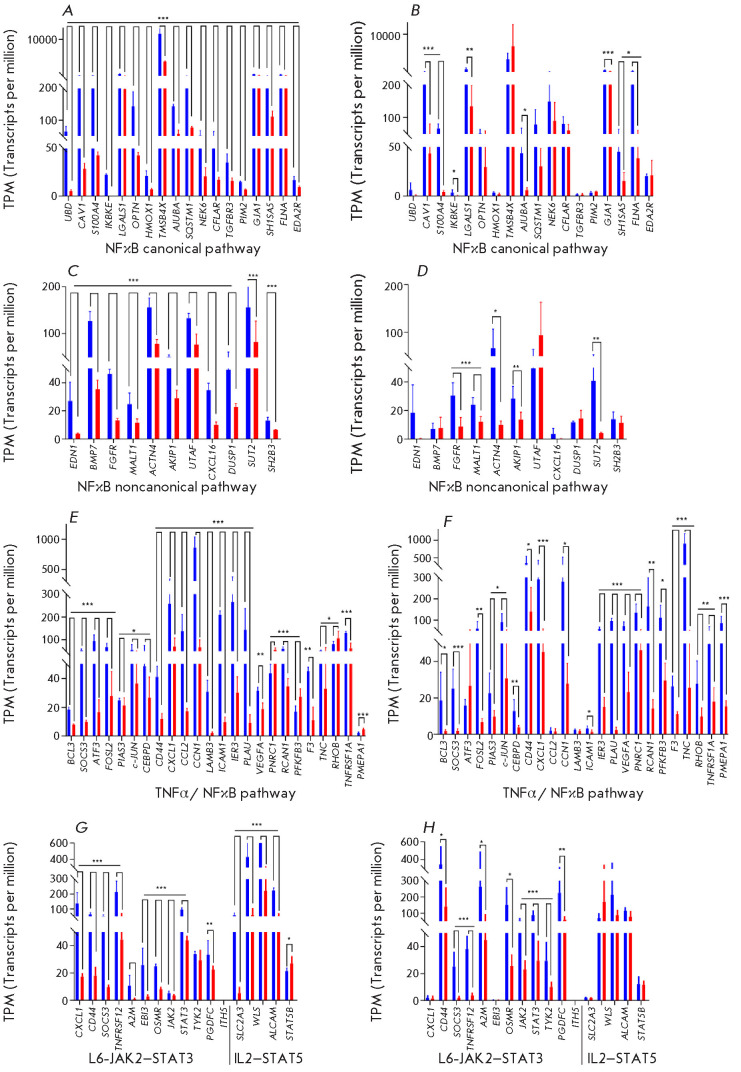
...

**Fig. 2:**
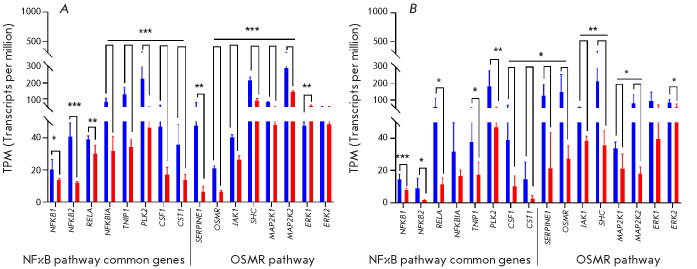
...

The procedures used for obtaining NPs and glial cell lines, as well as preparing the RNA-seq, have been described previously [25, 26, 27]. *Table 1S *(*Supplement 1*) summarizes the characteristics of NPs and glial cell lines from healthy donors and PD patients. *Figures 1S and 2S *(*Supplement 1*) present the results of immunocytochemical staining of glial cells with antibodies against the astrocyte marker S100 and NPs, with antibodies against the marker SOX1, demonstrating a high representation level of the indicated cell types. RNA-seq of NPs was performed as described in ref. [25]. RNA-seq of glia was performed using the Illumina NovaSeq 6000 technology.

**Real-time PCR analysis**

Quantitative RT-qPCR was performed using the procedure described in ref. [27]. The primers used in this study are listed in *Table 2S *(*Supplement 1*).

**Bioinformatics analysis**

Read mapping from the RNA-seq data was performed according to the procedure described in [25]. DEGs were identified according to the number of reads using the edgeR package as described in [27]; restrictions on DEGs with a significance of Pval < 0.05 were used for further analysis. The significance of the gene series FDR (False Discovery Rate) and Pval was determined using the GSEA method [28]. The Hallmark50 (UC San Diego) signaling pathways and GO (gene ontology) categories (http://gsea-msigdb.org) [29] were analyzed by the GSEA method (Analysis of Gene Sets) [28] using the computing capabilities and resources (http://www.webgestalt.org). Signaling pathways with FDR < 0.05 and Pval < 0.05 were selected. The multiple t-test was used to assess the significance of DEGs by the number of TPM [30].

## RESULTS AND DISCUSSION

**Comparative analysis of the NP and glia cell transcriptomes of PD patients carrying mutations in the *PARK2 *gene against healthy donors**

Several DEGs are simultaneously involved in a number of the enriched pathways identified by us (*Fig. 2*).

A large set of genes encoding proteins of the canonical NFκB, noncanonical NFκB signaling pathway, and the TNFα/NFκB proinflammatory signaling pathway is distinguishable among the DEGs (*Fig. 1* and *Fig. 2*), which includes, in particular, the genes en coding subunits of the NFκB transcription factor (*NFKB1, NFKB2, *and *RELA*), the *NFKBIA *gene (NFκB inhibitor), the *SOCS3 *gene of the negative regulator of NFκB signaling pathways, the inflammation suppression gene *PIAS3*, the *CCL2, CXCL1, *and* ICAM1 *chemokine genes responsible for cell adhesion, the *PLAU *and *PLK2 *genes involved in protein processing, the *PDGFC *and *VEGFA *genes encoding growth factors, the *PFKFB3 *gene controlling glycogenesis, and the *TGFB2 *gene of the transforming growth factor. Expression of only the *PFKFB3 *and* PNRC1 *genes in the NP cells of PD patients is upregulated compared to the case in the cells of healthy donors (*Fig. 1*).

Therefore, it is worth mentioning that current concepts of the immune system response to various factors include the involvement of the inflammatory canonical, the non-canonical NFκB pathway, and the TNFα/NFκB signaling pathway [31, 32]. These pathways mediate the immune response through the synthesis of cytokines (interferons, interleukins, and chemokines). Upon infection, pro-inflammatory proteins of the NFκB family of the canonical pathway are activated via a disruption of the association between factors belonging to this family and the inhibitory complex: the movement from the cell cytoplasm to the nucleus and transcription of target genes. The TNFα/NFκB, IL6–STAT3 signaling pathways can be activated during the non-canonical response of the immune system to the emergence of cytokines: TNFα in particular.

Downregulated expression of the inhibitory genes* SOCS3, NFKBIA *[33, 34], and *PIAS3 *[35] suggests that a dynamic development of the immune system response associated with NFKB signaling pathways over time is possible.

IL6 is known to be the target gene of the NFκB signaling pathway and an inducer of the IL6-JAK2-STAT3 pathway, whose activation leads to inflammation at the organismal level. The expression of most genes involved in the IL6-STAT3 signaling pathway has been shown to be reduced both in NPs and glia of PD patients compared to HD cells (*Fig. 1G,H*). We observed a decrease in the expression of the IL2-STAT5 signaling pathway genes *ITIH5, ALCAM, SLC2A3, *and *WLS *and an increase in the expression of the *STAT5B *gene in NP cells of PD patients compared to the NPs of healthy donors; however, expression of the genes involved in this pathway in glial cells remained unchanged (*Fig. 1G,H*).

**Fig. 3:**
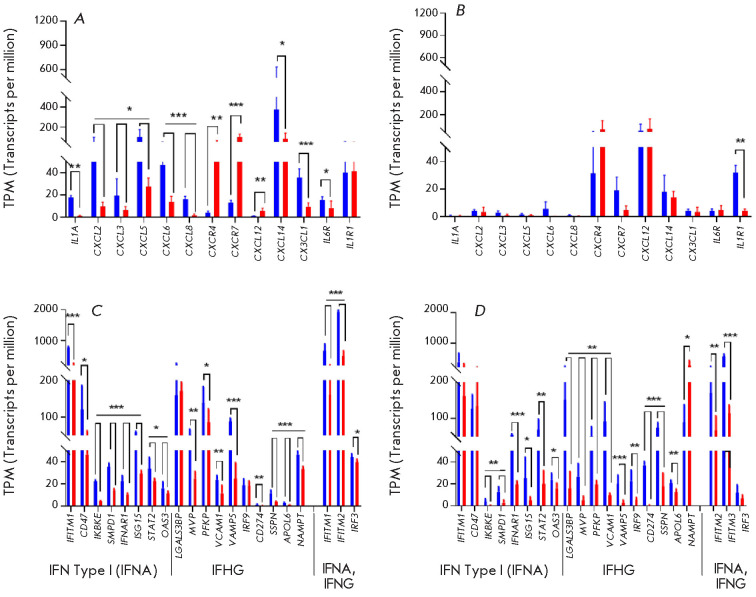
...

In PD patients, a large set of genes encoding proteins of the cellular response to the interferons IFNα and IFNγ (*Fig. 3 C,D*) in NP and glial cells is also characterized by reduced expression compared to healthy donors. It is known that the expression of interferon and immune-related genes is controlled by interferon regulatory factors (IRFs). The targets of the *IRF3 *factor include *IFITM1-3 *genes. The products of *IFITM1-3 *genes are capable of blocking viral infections by altering membrane properties. The product of the *IFITM3 *gene exerts an inhibitory effect on a wide range of viruses, while the products of *IFITM1,2 *genes are characterized by a narrow functional specificity [22, 36, 37, 38, 39]. Activation of* IFITM1-3 *is associated with the signaling pathway connected to the membrane receptor OSMR [40]. *Figure 2*and *Figure 3* present the data for the genes involved in the OSMR signaling pathway (associated with PD [41]), *SHC *(the gene encoding the signal transduction adaptor protein), *JAK1, MAP2K1,* and *MAP2K2 *(phosphokinase genes), as well as other genes involved in the interferon response: *IFITM2,3, MVP, PFKP, VCAM1, VAMP5, TNFRSF1A, TYK2, *and* PDGFC*, which have significantly downregulated expression in the NP and glial cells of PD compared to healthy donors. The uncovered reduced expression of the genes involved in the OSMR signaling pathway can cumulatively weaken the transduction of the extracellular signal into the cell.

The gene encoding the transmembrane protein CD47 is involved in neuroprotection by astrocytes and other immune cells from the environment of degenerated DA neurons [42]. Reduced expression of the* CD47 *gene in the NPs of PD patients as compared to healthy donors may be indicative of neuroprotection weakening during the development of PD.

Furthermore, there is a decrease in the expression of the chemokine genes *CXCL5, CXCL6, *and* CXCL8*, which are responsible for the chemotaxis of immune cells to the inflammation foci in the NP cells of PD patients when compared to healthy donors (*Fig. 3A, B*). Meanwhile, their receptor *CXCR2 *was not expressed in NPs. The *CXCL5 *gene is associated with PD [43], while the *CXCL6 *and *CXCL8 *genes are associated with PD via their effect on the differentiation of DA neurons [44]. Expression of some genes involved in the functioning of the immune system (*CXCL12, CXCR4, CXCR7*) in the NP cells of PD patients is upregulated compared to that in healthy donors (*Fig. 3A*). The altered expression of these genes is associated with the development of neurodegenerative diseases, including PD [45].

**Fig. 4:**
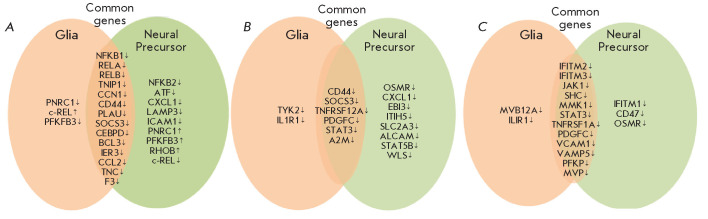
...

Hence, both in the NP cells and glia of PD patients, expression of a large number of DEGs related to innate and adaptive immunity (in particular, to inflammation) is downregulated. While there is a general similarity between NP cells and glia, their sets of DEGs do not completely match (*Fig. 4*).

**Fig. 5:**
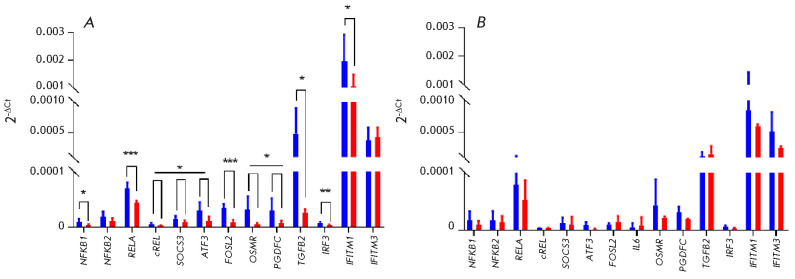
...

The RNA-seq data in NP cells was confirmed by RT-qPCR for selected genes (*Fig. 5A*). Similar trends exist in DEGs when assessed through RNA-seq and a RT-qPCR analysis in glial cells as well, but with a lower significance (*Fig. 5B*).

*Figure 3S *(*Supplement 1*) shows the data on the expression of gene series by Gene Ontology categories: inflammation and chronic inflammation (*A,B*), response to molecules of bacterial origin (*C,D*), binding to cytokines and cytokine receptors (*E,F*), and negative regulation of cytokine production (*G,H*).* Figure 4S *(*Supplement 1*) shows the data according to the Complement cascade category (*A,B*) of the HallMark50 resource. It is clear that in the PD lines, gene expression is predominantly reduced compared to that in healthy donors.

**The hypothetical mechanisms leading to disturbances in innate immunity functioning in NP cells of PD patients carrying mutations in the *PARK2 *gene**

Certain assumptions regarding the impact of mutations in the *PARK2 *gene on how immunity functions in PD patients can be made based on earlier data, and our findings. As a ubiquitin ligase, the native Parkin protein is involved in the ubiquitination of the IKBKG/IKKg/NEMO subunit, which is a component of the NFκB inhibitory complex in the cytoplasm [46]. This promotes the activation of the NFκB1 and RELA proteins and upregulation of the expression of inflammatory factors [47], including the NFκB factors *per se* due to the autoregulation of the target factors RELA (*Table 4S, Supplement 1*). It is fair to assume that the ability to ubiquitinate is not tapped if there is a mutation in the *PARK2 *gene, this resulting in a suppression of the NFκB factor activation in the lines of PD patients compared to healthy donors.

**Fig. 6:**
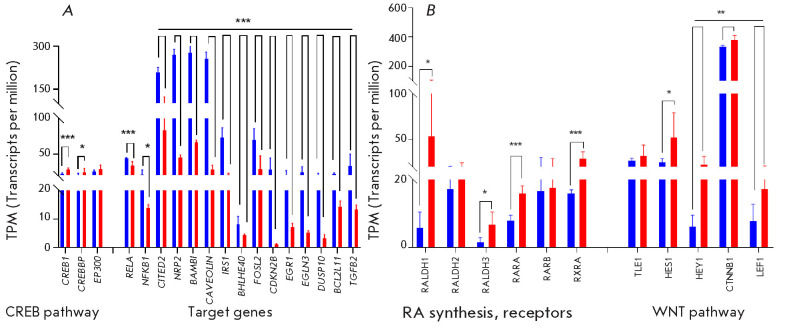
...

Previously, we revealed a significant increase in the expression level of many *HOX *genes in the NP cells of PD patients carrying mutations in the *PARK2 *gene compared to healthy donors [27]. There is evidence that some HOX proteins can inhibit CREBBP/CBP acetyltransferase activity [48]. The CREB transcription factor and the associated signaling pathway (CREB – CREBBP/CBP and/or EP300) are known to play an important role in the immune system [49]. We analyzed the RNA-seq data on the expression of the genes involved in the CREB signaling pathway in NP and glial cells, as well as a number of the target genes of this signaling pathway identified upon determination of the CREB regulon in the human genome using various methods [50, 51] and in relation to stress, transcription, and immune system signaling pathways (*Fig. 6*). Expression of CREB pathway genes is virtually identical in the glia of PD patients and is slightly elevated in the NP cells of PD patients, whereas expression of the target genes is significantly downregulated in NPs of PD patients compared to healthy donors. It is possible that the upregulated expression of HOX genes in the NP lines of PD patients compared to healthy donors could indirectly lead to a downregulation of the expression of the target genes in the CREB signaling pathway (CREBBP–HOX genes), target genes [27],* RELA, *and *NFKB1 *in particular.

Activation of *HOX *genes can be triggered by increased synthesis of retinoic acid (RA) [27]; accordingly, the mechanism of suppression of inflammation and NFκB expression in PD patients can be linked to RA [52]. Our analysis of the expression of the *RALDH1, RALDH2, *and *RALDH3 *genes associated with RA synthesis, as well as the genes of the nuclear receptors* RARA *and *RXRA *(*Fig. 6B*) and their activator* PNRC1 *(*Fig. 1E*), showed that the expression levels of these genes were higher in NPs.

It is also known that the Parkin protein normally has a stabilizing effect on the CTNNB1 factor (β1- catenin), as a co-activator of the transcription factor LEF1 [53]. Mutations in the *PARK2 *gene in PD patients can destabilize β1-catenin, affecting the functioning of the coupled complex, including the transcriptional repressors HES1 and HEY1. The factors* CTNNB1, TLE1, LEF1, HES1, *and *HEY1, *as part of the transcriptional complex [54, 55, 56, 57], can significantly suppress the expression of the target genes (*Table 4S, Supplement 1*). According to our findings, expression of the *CTNNB1, LEF1, HES1, *and *HEY1 *genes is upregulated in NP cells of PD patients compared to healthy donors (*Fig. 6*), which can presumably suppress the transcription of their target genes, including the transcription factors *BCL3, ATF3, JUN, *and* STAT3*.

**Fig. 7:**
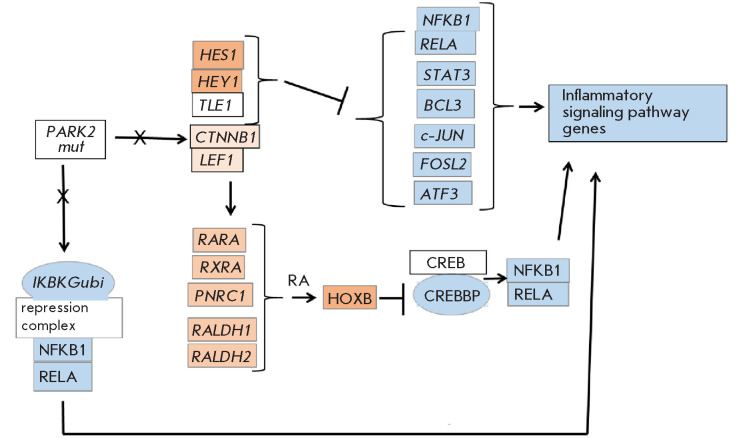
...

An analysis of the database of the target genes for transcription factors (http://maayanlab.cloud/harmonizome3.0) leads one to suggest that the revealed downregulated expression of many genes is rooted in the decreased expression of their transcription factors (*Table 4S, Supplement 1*), which depends on the ratio between pro- and anti-inflammatory factors, upregulated expression of the β-catenin-associated repressor group, and the lacking neuroprotection from the mutant Parkin protein.

*Figure 7* shows a hypothetical mechanism of the effect of *PARK2* dysfunction on the functioning of the immune-related genes in the NP cells of PD patients, which is a summary of the various possible signaling pathways. In the future, it will have to be determined which signaling pathway(s) are more important as relates to the Parkinson disease.

The conducted analysis of DEGs in NP and glial cells obtained from the iPSCs of PD patients indicates that the expression of the genes of the innate immune system is downregulated compared to that of healthy donors. It is noteworthy that NP and glial cells obtained as a result of directed differentiation of iPSCs* in vitro *are more likely to correspond to cells at the embryonic stage of development [58]. Hence, the observed decrease in the transcription level of the genes of the innate immune system in the cells obtained from PD patients carrying mutations in the *PARK2* gene compared to HD cells is presumably indicative of initial prodromal stages of PD development.

In this regard, it is also worth noting the existing data according to which the Parkin protein is an activator of innate immunity [59]. This fact can serve as indication that the absence of synthesis of the Parkin protein as a result of a gene mutation would lead to innate immunity suppression. This is what is observed in the NPs and glia of PD patients carrying the mutant* PARK2 *gene as uncovered in this study.

The ... 12

The ... 13

The ... 14

## EXPERIMENTAL PROCEDURES

The ... 1

The ... 2

The ... 3

The ... 4

The ... 5

The ... 6

The ... 7

The ... 8

The ... 9

The ... 10

The ... 11

The ... 12

The ... 13

The ... 14

## RESULTS

The ... 1

The ... 2

The ... 3

The ... 4

The ... 5

The ... 6

The ... 7

The ... 8

The ... 9

The ... 10

The ... 11

The ... 12

The ... 13

The ... 14

## DISCUSSION

The ... 1

The ... 2

The ... 3

The ... 4

The ... 5

The ... 6

The ... 7

The ... 8

The ... 9

The ... 10

The ... 11

The ... 12

The ... 13

The ... 14

The ... 1

The ... 2

The ... 3

The ... 4

The ... 5

The ... 6

The ... 7

The ... 8

The ... 9

The ... 10

The ... 11

The ... 12

The ... 13

The ... 14

## CONCLUSIONS

A large group of genes with decreased expression was identified in NP and glial cell lines from PD patients when compared to healthy donors. These genes are for the most part involved with the innate immune system signaling pathways NFκB, IL6-STAT3, IL2-STAT5, IFNα, IFNγ and the cellular response to the stress signaling pathway CREB. Only a limited number of immune-related genes was found to be overexpressed in NP cells from PD patients. There are many common immune-related genes with decreased expression in both the NP and glial cells of PD patients compared to healthy donors. Among them are the genes encoding pro-inflammatory factors (*NFKB1 *and *RELA*), immune system suppressors (*NFKBIA*, *SOCS3*, and *PIAS3*), components of the IL6- STAT3 signaling pathway (*JAK1 *and *STAT3*), as well as components of the OSMR signaling pathway. The genes of the anti-inflammatory complexes associated with retinoic acid production and characterized by increased expression compared to healthy donors were identified in the NP cells of PD patients.

The expression of a number of the genes responsible for adhesion (*CCL2, CXCL1, *and *ICAM1*), lymphocyte migration to the sites of inflammation (*CXCL2, CXCL5, CXCL6, *and *CXCL8*), maintenance of endothelial and epithelial proliferation (*PDGFC, VEGFA,* and *HBEGF*), protein processing (*PLAU *and *PLK2*), as well as energy exchange between astrocytes and neurons (*PFKFB3*) and communication between them (*CD47*) is downregulated in the NP and glial cells of PD patients compared to those of healthy donors.

## Abbreviations

PD: Parkinson’s disease
HD: healthy donors
DA neurons: dopaminergic neurons
DEG: differentially expressed gene
iPSCs: induced pluripotent stem cells
NP: neural precursors
PCR: polymerase chain reaction
ECM: extracellular matrix
IFN: interferon
IRF: interferon-regulated factors
RNA-seq: whole transcriptome sequencing
TPM: transcripts per million of mapped fragments
